# Disease Activity, Inflammation Markers, and Quality of Life Are Associated with Muscle Strength in Croatian Rheumatoid Arthritis Patients—A National-Based Study

**DOI:** 10.3390/medicina60091406

**Published:** 2024-08-28

**Authors:** Mislav Radić, Ivan Vlak, Marijana Vučković, Senka Rendulić Slivar, Mira Kadojić, Doris Stamenković, Dubravka Bobek, Josipa Radić, Andrea Gelemanović, Andrej Belančić, Erim Bešić, Tonko Vlak

**Affiliations:** 1 Internal Medicine Department, Rheumatology, Allergology, and Clinical Immunology Division, Center of Excellence for Systemic Sclerosis in Croatia, University Hospital of Split, 21000 Split, Croatia; mislavradic@gmail.com; 2 Department of Internal Medicine, School of Medicine, University of Split, 21000 Split, Croatia; 3 Institute of Physical Medicine and Rehabilitation with Rheumatology, University Hospital Split, 21000 Split, Croatia; ivst.05@hotmail.com (I.V.); tonkovlak@gmail.com (T.V.); 4 Internal Medicine Department, Nephrology and Dialysis Division, University Hospital Split, 21000 Split, Croatia; mavuckovic@kbsplit.hr; 5 Toplice Lipik, Special Hospital for Medical Rehabilitation, The Teaching Base of School of Medicine University of Osijek, 34551 Lipik, Croatia; senka.rendulic@bolnica-lipik.hr; 6 Institute of Physical Medicine and Rehabilitation, University Hospital Osijek, 31000 Osijek, Croatia; m.agata@kbo.hr; 7 Department of Physical and Rehabilitation Medicine, Clinical Hospital Centre Rijeka, 51000 Rijeka, Croatia; stamenkovicdoris@gmail.com; 8 Institute of Physical Medicine and Rehabilitation with Rheumatology, Dubrava University Hospital Zagreb, 10000 Zagreb, Croatia; dubravka.bobek@hotmail.com; 9 Mediterranean Institute for Life Sciences (MedILS), 21000 Split, Croatia; andrea.gelemanovic@gmail.com; 10 Department of Basic and Clinical Pharmacology with Toxicology, Faculty of Medicine, University of Rijeka, Braće Branchetta 20, 51000 Rijeka, Croatia; andrej.belancic@uniri.hr; 11 Faculty of Pharmacy and Biochemistry, Department of Biophysics, University of Zagreb, 10000 Zagreb, Croatia; ebesic@pharma.hr; 12 Department of Physical and Rehabilitation Medicine, School of Medicine, University of Split, 21000 Split, Croatia

**Keywords:** rheumatoid arthritis, disease activity, muscle strength

## Abstract

*Background and Objectives:* Rheumatoid arthritis (RA) patients experience sarcopenia and decreased muscle mass and handgrip strength, leading to decreased quality of life and disability. The prevalence of RA varies across regions. This study aimed to evaluate the factors associated with RA in Croatian regional centres and explore correlations between clinical parameters and muscle strength. *Materials and Methods:* Included in this study were 267 stable RA patients from four Croatian clinical centres. The patients’ mean age was 60.4 ± 12.0 years, with 12.7% of them being male. For each study participant, information was gathered on their anthropometric characteristics, clinical and laboratory indicators, quality of life, disease activity, and sociodemographics. *Results:* The main results showed that in the female RA participants, the significant positive predictors are weight, height, exercise, VAS, and haemoglobin level. The negative predictors are the use of conventional synthetic disease-modifying anti-rheumatic drugs, the use of biological disease-modifying anti-rheumatic drugs, the number of tender joints, the number of swollen joints, the estimated sedimentation rate, the C-reactive protein, the disease activity score, the parameters of the EQ5D, and being prescribed with three or more medications. In the male RA participants, significant predictors of muscle strength are only weight, height, and anxiety/depression difficulties, according to the EQ5D. *Conclusions:* This study showed correlations between muscle strength and the parameters of disease activity, inflammation parameters, health-related quality of life, therapy, and exercise in the female RA participants in Croatia.

## 1. Introduction

Rheumatoid arthritis (RA) is a chronic, systemic, autoimmune disease of unknown aetiology characterised by articular and extra-articular manifestations leading to functional disabilities and a huge socioeconomic and medical burden on society. This is the most commonly diagnosed systemic inflammatory arthritis with typical inflammatory changes in the synovial membrane of the mainly small, but also other-sized, joints. RA patients have a higher cardiovascular risk and show higher rates of depression, osteoporosis, obesity, sarcopenia, and infections. Depending on the presence of autoantibodies (rheumatoid factor and antibodies against post-translational modified proteins like citrullination—ACPA—and carbamylation—antiCarP—antibodies) RA can be defined as seropositive or seronegative RA [1,2,3,4].

Although the exact aetiology is still unknown, it is believed that genetic and environmental causes play a key role in the development of the disease. Genetical susceptibility is described as an increased risk of developing RA if a person has inherited the specific HLA-DRB1 alleles (especially DR4 and DR1), which show the most association with seropositive cases. It is also known that a positive family history increases the risk for RA 3–5 times [1,3,5]. The most notable environmental factor is smoking, while alcohol and coffee consumption, obesity, and a lower socioeconomic status and educational level may play a role in the potential onset of the disease and, later, its activity [2,4,5,6,7]. Microbial infections have also been connected with the development and, later, the exacerbation of RA. Clinical and animal model studies have suggested that infections by some microorganisms contribute to the etiopathogenesis of RA, of which *Porphyromonas gingivalis* exhibited the strongest associations; however, larger studies are needed to confirm these ideas [8].

Studies on different populations have shown the constant prevalence of RA at 0.5–1.0%. In the U.S. population, the prevalence is approximately 1%, with some exceptions in the Native American populations (e.g., Prima and Pagago Indians show a prevalence of 5.3%, while Chippewa Indians show the highest prevalence of 6.8%). The prevalence is lowest in North Africa, the Middle East, and Asia (0.16%), while Western European countries and the Australian population have a slightly higher prevalence (0.44% and 0.46%, respectively) [9,10]. A systematic review of six WHO regions showed female predominance and that there was no difference between urban and rural settings, with the estimation of growth in prevalence in the future [11]. The sex ratio is from 2:1 to 3:1 for women in comparison to men, and some studies also show higher disease activity in women. Nulliparity is associated with an increased risk of disease development whereas pregnancy seems to decrease this, and in most patients, the clinical symptoms are less expressed during pregnancy. Relapses of the disease are frequent in the postpartum period [9,12,13].

Although there is not much prior research in this area, what we have located describes a comparable prevalence of 0.46%, with a female predominance. The prevalence of RA in Croatia was 0.38% in a multi-country survey study conducted in Central and Eastern Europe between March 2009 and March 2010, which is comparable to the other European nations. During the specified period, the prevalence was reported to be 0.37% in Hungary, 0.61% in the Czech Republic, and 0.46% in Estonia.

In one study conducted in south Croatia on 869 RA patients from June 2013 to August 2015, Split-Dalmatia County showed a prevalence of 0.24% in adult patients with the Sinj region having the highest one, which was explained by a higher incidence of HLA-DRB1*04 in those patients. In addition, the RA population is characterised by a lower socioeconomic status [14,15,16]. There is a need for future RA studies to define the characteristics of the RA population in Dalmatia and Croatia [15].

Over the years, there has been an increase in RA prevalence and incidence in women but only in its prevalence in men, which has been interpreted as being because of the increase in life expectancy, the environmental factor effects, and because of better diagnostic and therapeutic treatments developing through the years among patients with RA [17].

The disease activity score (DAS) index, of which DAS-28 is most frequently employed, is the most popular method used to monitor RA patients [18,19]. Many studies have shown that RA patients have a higher frequency of sarcopenia, lower muscle mass and handgrip strength, and changes in body composition in favour of fat tissue. There is a lower quality of life in those patients because of the acute and chronic changes to the joints that lead to deformities, disability, and lower physical activity [20,21,22]. Lower handgrip strength was associated with lower quality of life, leading to less everyday physical activity and higher levels of pain and discomfort [23]. These factors depend on disease activity since in the patients with higher disease activity scores (DAS-28 and HAQ-DI), they are more significant. Smoking was also one of the factors correlated with the worsening of skeletal muscle density in patients [24,25]. With today’s modern radiological methods (magnetic resonance imaging and ultrasound) and functional tests, we can detect a positive correlation between joint inflammation and functional disability [26]. Although RA is a chronic condition, there are now efficient pharmaceutical therapy options that reduce inflammation, joint damage, and overall disease activity. Nevertheless, a patient’s physical function or strength is never fully restored [27].

The aim of this study was to assess the determinants of RA in the regional centres in Croatia and to investigate possible associations between muscle strength and clinical parameters.

## 2. Materials and Methods

This national study was carried out on the initiative of the Croatian Society of Physical and Rehabilitation Medicine. This nationwide study included 267 stable RA patients and was conducted at four centres in Croatia in 2017. In total, 134 participants were recruited from the Lipik centre, 72 participants were recruited from the Split centre, 35 participants were recruited from the Osijek centre, and 26 participants were recruited from the Rijeka centre.

RA diagnosis was made using the criteria of the 2010 American College of Rheumatology/European League Against Rheumatism classification [28].

All measurements for a single participant were performed on the day he/she was screened. Participants were screened on different days during the recruitment period.

**Figure 1:**
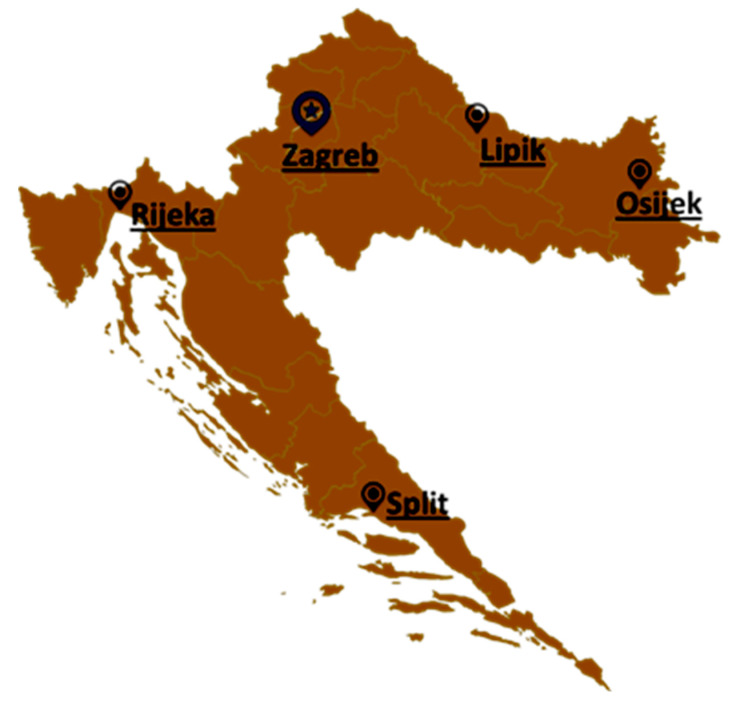
Geographical locations of clinical centres in Croatia which were included in this study.

The geographical locations of centres are represented in Figure 1.

We excluded patients who were minors, had active infection, had serious cognitive impairment, had overlap syndromes, or were unwilling to participate.

### 2.1. Clinical and Lab Specifications

Data on the length of RA illness, the existence of arterial hypertension, diabetes mellitus, chronic renal disease, hyperlipidaemia, and stomach ulcers were gathered for each participant by carefully reviewing their medical history. The following groups of medications were identified based on data collected on medication usage: NSAIDs, paracetamol/tramadol, corticosteroids (CSs), conventional synthetic disease-modifying anti-rheumatic drugs (CSDMARDs), biological disease-modifying anti-rheumatic drugs (BDMARDs), and the usage of three or more medications.

Data on haemoglobin, C-reactive protein (CRP), and erythrocyte sedimentation rate (ESR) were gathered in relation to laboratory parameters.

### 2.2. Sociodemographic Information

Data on marital status were gathered using a sociodemographic questionnaire. Information about the kind, length, and frequency of exercise was also gathered for every research subject.

### 2.3. Disease Activity and Quality-of-Life Assessment

The disease activity score (DAS28) is a measurable disease activity indicator that is displayed as a total score. It is derived from the number of sensitive joints, the number of swollen joints, the ESR, and a general health assessment (VAS).

Each participant’s health-related quality of life (hrQoL) was measured using the EQ5D-3L questionnaire. The five dimensions that make up the EQ-5D-3L descriptive system are mobility, self-care, regular activities, pain/discomfort, and anxiety/depression. There are three levels for each dimension: minimal issues, moderate issues, and severe issues, depicted on a 1–3 scale. By checking the box next to the statement that best fits each of the five dimensions, the patient is asked to identify the state of his or her health. The level chosen for that dimension is expressed as a 1-digit number as a result of this decision.

The EQ VAS, named for the Visual Analogue Scale it includes, is the second section of the questionnaire. This represents the respondent’s overall evaluation of their health on a range of 0 (the poorest possible health) to 100 (the finest possible health) [29].

### 2.4. Muscle Strength Assessment

Handgrip strength was assessed using a hand-held dynamometer (Saehan, Masanhoewon-gu, Changwon-si, Gyeongsangnam-do, Republic of Korea) for every study participant. Three measurements were taken, and average values were calculated.

### 2.5. Statistics

The Shapiro–Wilk test was performed to assess the normality of the continuous data. If the continuous data followed a normal distribution, they were presented with means and standard deviations (SDs); otherwise, medians and interquartile ranges (IQRs) were used. Categorical data were presented as numbers with percentages. To examine the difference among the three groups (remission + low disease activity vs. moderate disease activity vs. high disease activity), a chi-square test was used for the categorical data, whereas a one-way ANOVA or Kruskal–Wallis test was used for continuous data depending on the normality distribution. Due to the large variation in the number of male and female participants, a complete statistical analysis was conducted separately for each gender.

To identify independent predictors of muscle mass in RA patients, a generalised linear model was used, adjusted for age, BMI, and the location of the clinical centre. Results of the generalised linear models were presented with beta coefficients and standard errors. Statistically significant results were those with a *p*-value < 0.05. The entire statistical analysis was performed using the free software environment for statistical computing, R version 4.0.0.

## 3. Results

In this cross-sectional study, 267 RA patients were included; the mean age was 60.4 ± 12.0 years and 12.7% of the patients were male. The average time that the RA illness persisted was 15.1 ± 11.3 years.

Based on their DAS-28 scores, participants were split into three groups: remission combined with low disease activity (DAS-28 score < 3.3), moderate disease activity (DAS-28 score from 3.3 to 5.1), and high disease activity (DAS-28 score > 5.1).

Table 1 presents the differences in the measured parameters related to the disease activity for the female and male participants, respectively.

Table 2 and Table 3 display the beta coefficients for the effect of several independent predictors on muscle strength in the RA participants, adjusted for age, BMI, and the location of the clinical centre (only statistically significant values are shown) for female and male participants, respectively.

In the female RA participants, the significant positive predictors are weight, height, exercise, VAS, and Hb. The negative predictors are the use of BDMARDs, the use of CSDMARDs, the number of tender joints, the number of swollen joints, the ESR, CRP, the DAS, the parameters of the EQ5D, and being prescribed with three or more medications. In the male RA participants, the significant predictors of muscle strength are only weight, height, and anxiety/depression difficulties according to the EQ5D.

**Figure 2:**
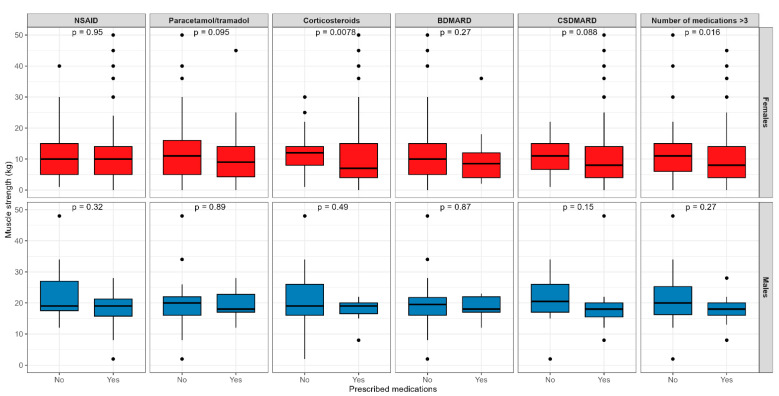
Differences in muscle strength regarding the different medication prescriptions in Croatian rheumatoid arthritis participants. **Abbreviations:** NSAID—non-steroid anti-inflammatory drug, BDMARD—biological disease-modifying anti-rheumatic drug, CSDMARD—conventional synthetic disease-modifying anti-rheumatic drug.

Figure 2 presents differences in muscle strength regarding the different medication prescriptions.

The only significant difference found was that the female RA participants prescribed with CS therapy and those prescribed with more than three medications had a lower level of muscle strength.

**Figure 3:**
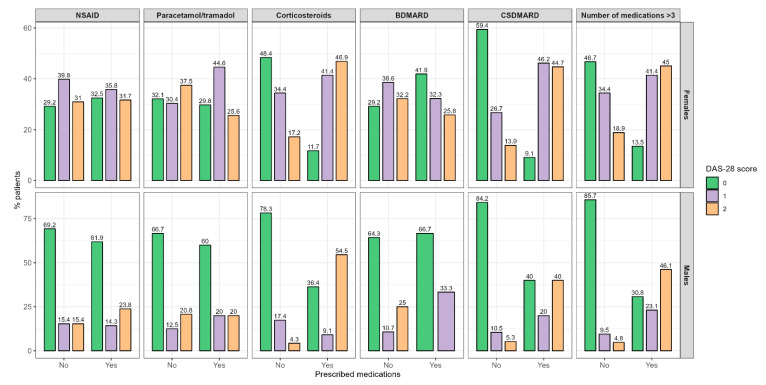
Disease activity differences regarding the prescription of different medication groups. **Abbreviations:** DAS 28—disease activity score 28, NSAID—non-steroid anti-inflammatory drug, BDMARD—biological disease-modifying anti-rheumatic drug, CSDMARD—conventional synthetic disease-modifying anti-rheumatic drug. 0—remission + low disease activity, 1—moderate disease activity, 2—high disease activity.

To determine the differences in disease activity regarding the RA therapy, we divided participants into the three mentioned stages of disease activity regarding the DAS-28 score and compared the differences regarding the prescription of different medication groups, which is shown in Figure 3.

A significant difference was found for the CS and CSDMARD therapies in males and females. The CS were more frequently prescribed in higher stages of disease activity in both males and females (*p* = 0.003 and *p* < 0.001, respectively). On the other hand, CSDMARDs in females were prescribed in higher disease activity stages (*p* < 0.001); meanwhile, in the male participants, they were equally prescribed in the lowest and highest disease activity groups (*p* = 0.018).

Also, the prescription of more than three drugs was significantly more common in the higher disease stages (*p* = 0.003 and *p* < 0.001 in males and females, respectively).

## 4. Discussion

This study showed correlations between muscle strength and the parameters of disease activity, inflammation parameters, health-related quality of life, therapy, and exercise in female RA participants in Croatia. The age difference may account for the differences in the different stages of disease activity identified in our study, including a higher BMI, more prescribed drugs, and a higher frequency of comorbidities. A regression analysis reduced this confounding effect.

The DAS-28 scores and the CRP and ESR values were found to be negative predictors of muscle strength in female RA participants. The number of swollen and tender joints was also associated with lower muscle strength. These results suggest an association between inflammation, muscle strength, and disease activity, but unfortunately, we were unable to establish causal relationships due to the study design. These findings are consistent with the results of a study of 199 Finnish RA patients, which found that the decrease in the patients’ composite score for muscle performance was linearly related to the increase in the DAS28 activity level [30]. This was also the case in a study of 50 RA patients from Egypt, which found that the DAS28-ESR score was significantly correlated with muscle performance tests, physical activity level, and fatigue score [31]. A prospective study on 107 RA participants showed that factors affecting muscle density decline include female sex, disease activity, smoking, and lower insulin-like growth factor 1 levels. Greater muscle density reduces annual worsening and clinically important worsening in HAQ scores and walking speeds [25].

Regarding inflammatory markers, the results of a review by Tuttle et al. show a strong correlation between higher levels of circulating inflammatory markers and lower skeletal muscle strength and muscle mass [32]. Another meta-analysis also confirmed a significant inverse correlation between the CRP and hs-CRP concentrations and muscle strength [33].

For all the parameters of the EQ5D—mobility, self-care, usual activities, pain/discomfort, and anxiety/depression—difficulty was found to be a negative predictor of muscle strength in the female RA participants along with VAS, as patients’ self-rated health.

The data on associations between quality of life and muscle strength in RA patients are rather scarce.

A study on 2365 arthritis patients from Korea found significantly higher odds ratios for the impairment of the mobility, usual activity, self-care, and pain/discomfort dimensions in RA patients with weak handgrip strength than those with normal handgrip strength [23]. In a study with 289 RA participants, Wiegmann et al. discovered that HAQ was an associated risk factor for falling [22]. When it comes to muscle strength and therapy associations, negative predictors of muscle strength in the women were the prescription of biological therapy and CSDMARDs as well as being prescribed three or more medications. This finding is probably related to the higher disease activity and a greater number of comorbidities in the patients treated with more drugs, which is supported by the results of our study depicted in Figure 2.

The use of CS therapy was not significant in our regression model, but we did find significant differences in females prescribed with CS therapy who showed lower levels of muscle strength than those not prescribed CSs. It is important to mention that our results also showed that CSs were more frequently prescribed in higher stages of disease activity in both females and males.

It has been shown that glucocorticoids induce oxidative stress in a variety of tissues, including muscle, nerve fibres, and bone. A common reaction to this type of stress is the up-regulation of the transcription factors classified as “anti-oxidants” in the forkhead box O family. This could be the reason for some of the hypodynamic/adynamic bone and muscle atrophy. It is more difficult to distinguish between physiological responses brought on by stress exposure and those brought on by inflammation as inflammatory reactions have access to the FOXO/anti-oxidant pathways [34]. It is important to highlight that when this study was conducted, JAK inhibitors were not approved for prescription for this indication in Croatia as per the National Health Insurance reimbursement criteria.

Interestingly, a positive predictor of muscle strength in female RA participants in Croatia was exercise, but no significant association was found between muscle strength and the type, frequency, or duration of exercise. The possible bias in our study could be that the data about exercise were collected by self-administered questionnaires.

It is important to remember that exercise in any form does not worsen the ESR or disease activity, suggesting that exercise is generally safe for people with RA. Further research is needed as the data on the type and duration of exercise that are useful for patients with RA are inconclusive [35].

The anthropometric parameters of height and weight were significant positive predictors of muscle strength in both male and female RA participants, which is self-explanatory.

Other than those mentioned, the only significant predictor of muscle strength in the male RA participants was anxiety/depression difficulties based on the EQ5D. The discrepancy of significant predictors of muscle strength regarding sex could be due to the small number of male participants in our study, and these results should be interpreted in such a way.

The cross-sectional design of this study limits our ability to determine causal relationships, which is the main source of this study’s limitations. Additionally, the self-reported questionnaire used to measure physical activity may introduce bias into the interpretation of the findings.

## 5. Conclusions

The findings of this nationally based study on RA in Croatia provide important new information about the clinical factors associated with RA in this context. Relationships have been found between muscle strength and physical functioning, therapy, inflammatory indicators, quality of life, and disease activity.

## Figures and Tables

**Table 1:** Differences in the measured parameters related to the disease activity.

	Females				Males			
	Remission + Low Disease Activity (N = 72)	Moderate Disease Activity (N = 88)	High Disease Activity (N = 73)	*p*	Remission + Low Disease Activity (N = 22)	Moderate Disease Activity (N = 5)	High Disease Activity (N = 7)	*p*
Disease duration (years), median (IQR)	12 (13.5)	14 (13)	12 (12)	0.867	10.5 (11)	15 (14)	7 (9.5)	0.645
Age (years), median (IQR)	54.5 (20)	63 (12)	68 (16)	<0.001	56 (13)	63 (9)	64 (12)	0.071
Weight (kg), median (IQR)	67.5 (15.4)	73.85 (16.72)	72 (18)	0.068	82.85 (10.12)	89 (3.6)	75 (9.4)	0.149
Height (cm), mean (SD)	165.39 (6.21)	163.89 (6.93)	163.62 (5.92)	0.097	177.95 (5.42)	173 (7.68)	174.43 (7.59)	0.124
BMI (kg/m^2^), median (IQR)	24.66 (6.48)	27.23 (7.02)	27.12 (6.14)	0.013	26.25 (5.5)	29.74 (3.6)	25.2 (3.17)	0.185
Muscle strength (kg)	12 (7)	10 (10)	6 (9)	0.002	20 (9)	16 (9.25)	17 (3.5)	0.155
Exercise (yes), N (%)	62 (86.11)	57 (65.52)	36 (49.32)	<0.001	16 (72.73)	2 (40)	6 (85.71)	0.215
Arterial hypertension, N (%)	22 (30.56)	43 (48.86)	46 (63.01)	<0.001	8 (36.36)	3 (60)	4 (57.14)	0.465
Neurological disease, N (%)	37 (51.39)	17 (19.32)	9 (12.5)	<0.001	9 (40.91)	/	1 (14.29)	0.119
Diabetes mellitus, N (%)	2 (2.78)	9 (10.23)	9 (12.33)	0.095	1 (4.55)	3 (60)	2 (28.57)	0.009
Dyslipidaemia, N (%)	7 (9.72)	14 (15.91)	15 (20.55)	0.194	2 (9.09)	4 (80)	1 (14.29)	0.002
Kidney disease, N (%)	3 (4.17)	3 (3.41)	3 (4.11)	0.961	/			
Gastric ulcer disease, N (%)	4 (5.56)	14 (15.91)	16 (21.92)	0.018	/	/	1 (14.29)	0.666
Liver disease, N (%)	2 (2.78)	1 (1.14)	2 (2.74)	0.709	1 (4.55)	/	/	0.666
Pulmonary disease, N (%)	2 (2.78)	8 (9.09)	1 (1.37)	0.046	1 (4.55)	1 (20)	/	0.315
Coronary artery disease, N (%)	2 (2.78)	8 (9.09)	9 (12.33)	0.101	/	1 (20)	1 (14.29)	0.432
Peripheral vascular disease, N (%)	3 (4.17)	6 (6.82)	4 (5.48)	0.767	/			
Inflammatory bowel disease, N (%)	1 (1.39)	2 (2.27)	1 (1.37)	0.878	/			
NSAID, N (%)	39 (54.17)	43 (48.86)	38 (52.05)	0.795	13 (59.09)	3 (60)	5 (71.43)	0.839
Paracetamol/tramadol, N (%)	36 (50)	54 (61.36)	31 (42.47)	0.053	6 (27.27)	2 (40)	2 (28.57)	0.852
Corticosteroids, N (%)	13 (18.06)	46 (52.27)	52 (71.23)	<0.001	4 (18.18)	1 (20)	6 (85.71)	0.003
BDMARD, N (%)	13 (18.06)	10 (11.36)	8 (10.96)	0.360	4 (18.18)	2 (40)	/	0.199
CSDMARD, N (%)	12 (16.67)	61 (69.32)	59 (80.82)	<0.001	6 (27.27)	3 (60)	6 (85.71)	0.019
Number of medications ≤3, N (%)	57 (79.17)	42 (47.73)	23 (31.51)	<0.001	18 (81.82)	2 (40)	1 (14.29)	0.003
Number of medications >3, N (%)	15 (20.83)	46 (52.27)	50 (68.49)	<0.001	4 (18.18)	3 (60)	6 (85.71)	0.003
Tender joint number, median (IQR)	0 (0)	6 (4)	14 (8)	<0.001	0 (2)	4 (8)	14 (8.5)	<0.001
Swollen joint number, median (IQR)	0 (0)	2 (3.25)	9 (4)	<0.001	0 (0)	2 (2)	6 (7)	<0.001
ESR (mm/h), median (IQR)	9 (6)	15 (10.25)	30 (26)	<0.001	8 (8.75)	14 (6)	13 (26.5)	0.021
DAS VAS, median (IQR)	10 (39)	50 (28.75)	50 (25)	<0.001	Too few data points			
DAS-28 score, median (IQR)	1.88 (0.75)	4.24 (0.82)	6.1 (0.98)	<0.001				
Mobility—no problem, N (%)	24 (33.33)	22 (25)	9 (12.33)	0.0482	5 (22.73)	1 (20)	2 (28.57)	0.932
Mobility—some problem, N (%)	47 (65.28)	63 (71.59)	61 (83.56)		17 (77.27)	4 (80)	5 (71.43)	
Mobility—extreme problems or unable to move, N (%)	1 (1.39)	3 (3.41)	3 (4.11)		/			
Self-care 1, N (%)	34 (47.89)	33 (37.5)	13 (17.81)	<0.001	9 (40.91)	2 (40)	3 (42.86)	0.388
Self-care 2, N (%)	37 (52.11)	47 (53.41)	49 (67.12)		13 (59.09)	3 (60)	3 (42.86)	
Self-care 3, N (%)	/	8 (9.09)	11 (15.07)		/	/	1 (14.29)	
Usual activities 1, N (%)	30 (41.67)	23 (26.14)	7 (9.86)	<0.001	7 (31.82)	2 (40)	4 (57.14)	0.187
Usual activities 2, N (%)	42 (58.33)	60 (68.18)	57 (80.28)		15 (68.18)	3 (60)	2 (28.57)	
Usual activities 3, N (%)	/	5 (5.68)	7 (9.86)		/	/	1 (14.29)	
Pain/discomfort 1, N (%)	24 (34.78)	6 (7.14)	1 (1.39)	<0.001	7 (31.82)	/	/	0.035
Pain/discomfort 2, N (%)	45 (65.22)	69 (82.14)	62 (86.11)		15 (68.18)	4 (80)	7 (100)	
Pain/discomfort 3, N (%)	/	9 (10.71)	9 (12.5)		/	1 (20)	/	
Anxiety/depression 1, N (%)	37 (56.92)	36 (42.35)	21 (29.17)	<0.001	12 (54.55)	3 (60)	4 (57.14)	0.728
Anxiety/depression 2, N (%)	28 (43.08)	44 (51.76)	46 (63.89)		9 (40.91)	1 (20)	2 (28.57)	
Anxiety/depression 3, N (%)	/	5 (5.88)	5 (6.94)		1 (4.55)	1 (20)	1 (14.29)	
Visual Analogue Scale, median (IQR)	70 (31.25)	59.5 (21.5)	49 (15)	<0.001	69.23 (16.05)	57.4 (21.16)	55.57 (12.43)	0.038
EQ5D index, median (IQR)	0.7 (0.18)	0.66 (0.18)	0.59 (0.21)	<0.001	0.71 (0.14)	0.6 (0.19)	0.66 (0.19)	0.315
CRP (mg/L), median (IQR)	2 (2)	3.9 (5.38)	7.4 (12)	<0.001	2 (1.88)	3.7 (0.4)	5.2 (10.86)	0.080
Hb (g/L), median (IQR)	134.5 (11)	132 (13.25)	128 (13)	0.003	147.41 (10.57)	135.2 (11.34)	137 (16.69)	0.029

**Abbreviations:** BMI—body mass index, NSAID—non-steroid anti-inflammatory drug, CSDMARD—conventional synthetic disease-modifying anti-rheumatic drug, BDMARD—biological disease-modifying anti-rheumatic drug, DAS 28—disease activity score, ESR—sedimentation rate, CRP—c-reactive protein, Hb—haemoglobin.

**Table 2:** Predictors of muscle strength in female rheumatoid arthritis participants.

Predictor	Beta	SE	*p*
Weight (kg)	0.295	0.085	<0.001
Height (cm)	0.243	0.077	0.002
Exercise (yes)	2.085	1.036	0.053
BDMARD	−3.207	1.419	0.024
CSDMARD	−2.511	1.178	0.034
Number of medications ≥ 3	−2.152	1.046	0.045
Number of tender joints	−0.242	0.074	0.001
Number of swollen joints	−0.362	0.106	<0.001
ESR (mm/h)	−0.091	0.030	0.003
DAS-28 score	−1.053	0.336	0.001
Mobility difficulties	−3.701	1.123	0.001
Self-care difficulties	−3.556	0.822	<0.001
Usual activities difficulties	−4.117	0.954	<0.001
Pain/discomfort level	−2.697	1.0878	0.014
Anxiety/depression difficulties	−2.384	0.882	0.007
EQ-5D VAS	0.077	0.027	0.005
EQ 5D index	12.744	2.550	<0.001
CRP (mg/L)	−0.075	0.035	0.031
Hb (g/L)	0.141	0.045	0.002

**Abbreviations:** CSDMARD—conventional synthetic disease-modifying anti-rheumatic drug, BDMARD—biological disease-modifying anti-rheumatic drug, DAS 28—disease activity score, ESR—sedimentation rate, CRP—c-reactive protein, Hb—haemoglobin.

**Table 3:** Predictors of muscle strength in male rheumatoid arthritis participants.

Predictor	Beta	SE	*p*
Weight (kg)	0.81091	0.20135	<0.001
Height (cm)	0.66743	0.21429	0.004
Anxiety/depression difficulties	−7.51868	3.27161	0.03

## Data Availability

The data are available from the corresponding author upon e-mail request.
